# Reducing unintended valgus deformity after supracondylar femoral derotational osteotomy: Influence of derotation amount and osteotomy method based on femoral antecurvature

**DOI:** 10.1002/ksa.70041

**Published:** 2025-09-09

**Authors:** Jisu Park, Hyunkwon Kim, Seonjin Shin, Tae Woo Kim, Moon Jong Chang, Dai‐Soon Kwak

**Affiliations:** ^1^ Department of Orthopedic Surgery SMG‐SNU Boramae Medical Center Seoul Korea; ^2^ Department of Anatomy, Catholic Institute for Applied Anatomy, College of Medicine The Catholic University of Korea Seoul Korea; ^3^ Department of Orthopedic Surgery Seoul National University College of Medicine, SMG‐SNU Boramae Medical Center Seoul Korea

**Keywords:** 3D simulation, coronal alignment, femoral antecurvature, femoral derotational osteotomy, patellar instability

## Abstract

**Purpose:**

The purposes of this study were threefold: (1) to evaluate the influence of femoral antecurvature on coronal alignment changes following supracondylar femoral derotational osteotomy (FDO); (2) to investigate the combined effects of derotation angle and osteotomy orientation in relation to femoral antecurvature and (3) to propose a practical strategy for minimising valgus deviation after FDO based sagittal femoral bowing.

**Materials and Methods:**

Sixty‐six cadaveric femoral computed tomography (CT) scans were analysed using three‐dimensional (3D) simulation. Femurs were classified into three groups based on the degree of antecurvature using the distal diaphyseal angle (DDA). Virtual surgery was simulated at 7 cm above the joint line using two osteotomy methods (shaft‐perpendicular and distal femur condylar line [DFC]‐parallel) and two derotation angles (10° and 20°). Mechanical lateral distal femoral angle (mLDFA) was measured before and after simulation. Multivariable and stratified regression analyses were performed.

**Results:**

Greater antecurvature led to larger mLDFA decrease, with more pronounced valgus shifts at higher derotation angles. Making DFC‐parallel osteotomy significantly reduced valgus change regardless of bowing (*β* = −0.331, *p* = 0.005). A significant interaction was found between DDA and derotation angle (*β* = 0.015, *p* < 0.001). The combination of DFC‐parallel osteotomy with 10° derotation provided the least valgus change (*R*² = 0.84). The allowable derotation angle to maintain <1° valgus shift decreased as DDA increased.

**Conclusion:**

Femoral antecurvature significantly affects coronal alignment after supracondylar FDO. Although the optimal target angle for derotation remains a surgical choice, understanding the relationship between sagittal bowing and coronal alignment can help tailor patient‐specific decisions. To minimise valgus shift after supracondylar FDO, aligning the osteotomy plane parallel to the distal femur condylar line can be effective. This method is simple and practical for standard surgical procedures.

**Level of Evidence:**

Level IV.

**Abbreviations**
DDA: distal diaphyseal angle
FDO: femoral derotational osteotomy
mLDFA: mechanical lateral distal femoral angle

## INTRODUCTION

Recurrent patellar dislocation is a multifactorial condition influenced by various anatomical and biomechanical factors, including trochlear dysplasia, patella alta and increased femoral anteversion [16]. Among these, excessive femoral anteversion is a significant risk factor for patellar instability and, if uncorrected, can compromise the outcomes of surgical interventions for recurrent dislocation [3].

Femoral derotational osteotomy (FDO) is a surgical technique used to correct excessive femoral anteversion and improve patellofemoral tracking [8, 14, 17]. However, supracondylar FDO may lead to unintended changes in coronal alignment, particularly valgus deformity [5, 9, 11]. Because valgus malalignment contributes to patellar instability, minimising this change during supracondylar FDO is essential for favourable surgical outcomes [15]. According to Paley et al., performing rotational osteotomy not perpendicular to the mechanical axis could lead to a change in coronal alignment [13]. Although achieving an osteotomy perpendicular to both coronal and sagittal mechanical axes would theoretically prevent angular deviation, it is rarely feasible intraoperatively. Clinical and simulation studies have suggested that femoral antecurvature, osteotomy level and degree of derotation influence postoperative coronal alignment [7, 11]. Nevertheless, there is a paucity of literature offering practical strategies to mitigate such changes during supracondylar FDO.

The purposes of this study were (1) to evaluate the influence of femoral antecurvature on coronal alignment changes following supracondylar FDO; (2) to investigate the combined effects of derotation angle and osteotomy orientation relative to femoral antecurvature and (3) to propose a practical strategy for minimising valgus deviation after supracondylar FDO based on sagittal femoral bowing. The authors hypothesised that (1) valgus shift would be aggravated by femoral antecurvature; (2) this valgus‐inducing effect would be amplified at higher derotation angles and influenced by the orientation of the osteotomy plane and (3) the optimal amount of derotation to minimise the valgus shift would vary depending on the femoral antecurvature, and adjusting osteotomy plane could mitigate this change.

## MATERIALS AND METHODS

**Figure 1:**
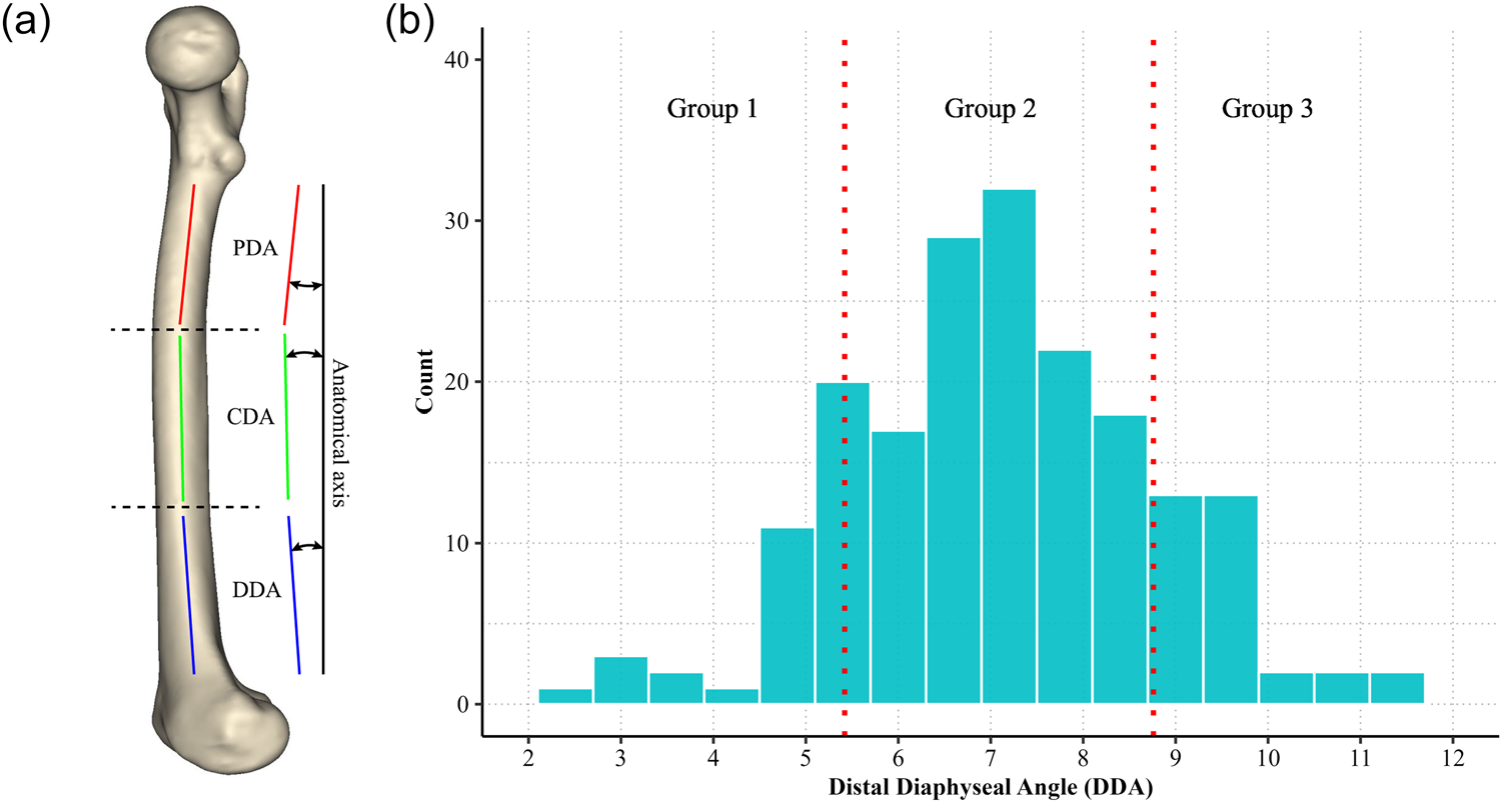
Measurement of femoral antecurvature and grouping based on distal diaphyseal angle (DDA). (a) Diagram illustrating measurement of the proximal diaphyseal angle (PDA), central diaphyseal angle (CDA) and DDA relative to the anatomical axis of the femur. (b) Histogram showing the distribution of DDA values in the study sample. Subjects were divided into three groups based on DDA.

Among 202 specimens from the Catholic Digital Human Library with precollected three‐dimensional (3D) computed tomography (CT) scans of cadaveric femurs, 66 femurs were utilised for the study. A total of 28 morphological parameters were extracted from each femur, including the degree of femoral sagittal bowing quantified using the distal diaphyseal angle (DDA) on the sagittal plane (Figure 1a) [1]. Based on the distribution of DDA values, samples were categorised into three groups, with cut‐off values of one standard deviation (SD) above and below the mean DDA (Figure 1b). Based on the cut‐off values (which were 5.5° and 8.8°), samples were randomly selected for each of the three groups (22 per group): Group 1 with the lowest DDA (lesser anterior bowing), Group 2 with average DDA, and Group 3 with the highest DDA (greater anterior bowing). Three‐dimensional reconstructions were performed using Mimics software (version 21; Materialise, Leuven, Belgium). The baseline radiologic measurements based on group are presented in Table 1.

**Table 1:** Baseline characteristics of each group.

	Group			*p*‐Value^c^
	1 (*N* = 22)	2 (*N* = 22)	3 (*N* = 22)	
Age^a^ (years)	46.9 (43–58)	51.1 (48–60)	53.1 (46–59)	0.235^c^
Height^b^ (cm)	162.3 ± 6.2	161.8 ± 6.6	162.5 ± 8.7	0.949^d^
Anteversion^a^ (°)	18.6 (9.1–24.1)	17.7 (9.4–25.1)	19.3 (14.1–27.8)	0.242^c^
DDA^a^ (°)	4.7 (3.5–5.0)	7.0 (6.9–7.1)	9.6 (9.3–10.1)	<0.001^c^
mLDFA^a^ (°)	85.4 (83.8–86.7)	84.7 (83.7–86.1)	84.1 (83.3–85.9)	0.072^c^

Abbreviations: DDA, distal diaphyseal angle; mLDFA, mechanical lateral distal femoral angle; N, number.

^a^ Values are expressed as median with interquartile range (Q1–Q3) in parentheses.

^b^ Values are expressed as mean with standard deviation in parentheses.

^c^ Kruskal–Wallis test.

^d^ Analysis of variance (ANOVA) test.

### Variable measurement

**Figure 2:**
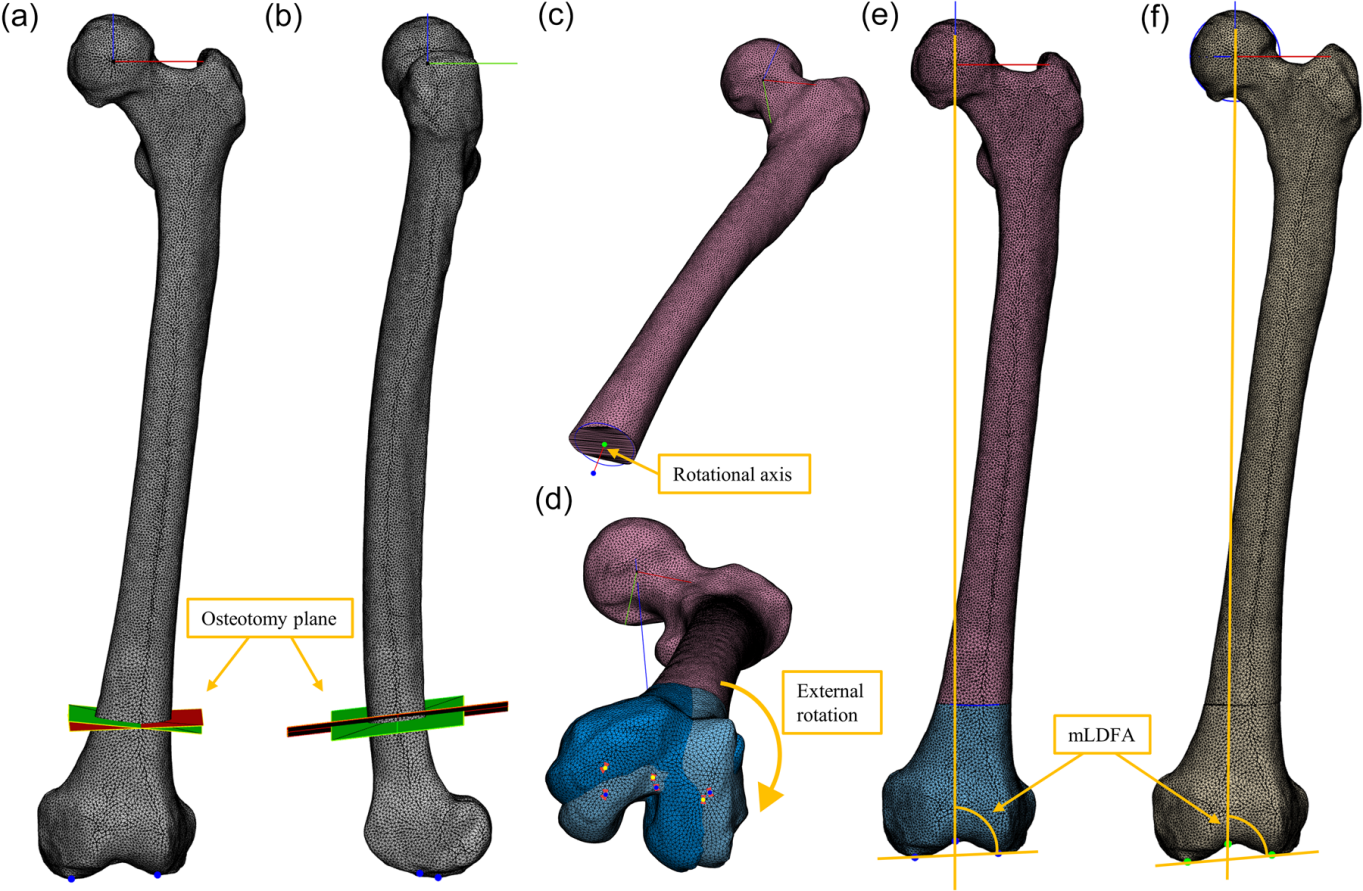
Supracondylar femoral derotational osteotomy (FDO) simulation and measurement of mechanical lateral distal femoral angle (mLDFA). (a) Osteotomy planes perpendicular to the anatomical axis of the distal femur (green) and parallel to the distal femur condylar line (red) are shown in coronal view. (b) Both osteotomy planes were perpendicular to the anatomical axis of the distal femur in sagittal view. (c) The rotation axis was set as the centroid of the cutting surface. (d) The distal fragment was externally rotated by 10° or 20°. (e) Measured mLDFA before supracondylar FDO. (f) Measured mLDFA after supracondylar FDO.

To assess coronal alignment change, the mechanical lateral distal femoral angle (mLDFA) was measured before and after simulated osteotomy (Figure 2). The hip centre was defined as the centre of a best‐fit sphere of the femoral head, and the distal femoral centre was identified at the intercondylar notch roof. The mechanical axis was defined as a line connecting these two points. The distal femur condylar line was established by linking the most distal points of the medial and lateral condyles in an orientation where the trochlear groove faced anteriorly. The mLDFA was the angle between the mechanical axis and the distal joint line in the coronal plane. After simulating surgery, the femoral mechanical axis was readjusted to be perpendicular to the ground, and the femoral trochlea was faced forward before remeasuring the mLDFA.

### Surgery simulation

Virtual surgery was performed using 3‐Matic software (version 13; Materialise). The osteotomy level was standardised to 7 cm proximal to the distal femur condylar line [5]. External rotation of the distal fragment was applied with the rotation axis centred on the centroid of the cutting surface (Figure 2).

### Osteotomy methods

Two osteotomy methods were simulated. The initial osteotomy plane was set perpendicular to the anatomical axis of the distal femur in both coronal and sagittal views, considering the actual surgical field (shaft‐perpendicular osteotomy). For the second method, the osteotomy plane was adjusted to become parallel to the distal femoral condylar line in the coronal view (DFC‐parallel osteotomy). Osteotomy plane adjustment for the second method was performed only in the coronal view (Figure 2a,b). The DFC‐parallel osteotomy was chosen because of the feasibility of landmark use in intraoperative fluoroscopy view.

### Derotation angle

To examine the change in mLDFA based on the degree of derotation, external rotation of the distal femur was set to 10° or 20°. These values correspond to common derotation amounts used to normalise femoral anteversion to physiological levels [17].

### Statistical analysis

Normality was assessed using the Shapiro–Wilk test. Group comparisons of mLDFA change were performed using the Kruskal–Wallis test. If significance was observed, the Mann–Whitney *U*‐test with Bonferroni correction was conducted. Within‐group comparisons (e.g., different derotation angles or osteotomy methods) were analysed using the Wilcoxon signed‐rank test. A multiple linear regression model was constructed to identify the independent and interactive effects of DDA, derotation angle, and osteotomy method on mLDFA change. Regression coefficients, 95% confidence intervals (CIs) and *R* ^2^ values were reported. In addition, stratified regression analysis was performed to determine the maximum allowable derotation angle producing <1° of valgus shift for each cutting method. All statistical analyses were conducted using R (version 4.3.3; R Foundation for Statistical Computing) and RStudio (version 2023.12.1 + 402; R Foundation for Statistical Computing).

## RESULTS

### Influence of femoral antecurvature on mLDFA change

Group‐based analysis revealed that valgus shift, represented by a decrease in mLDFA, was most prominent in the group with high femoral bowing and least in low femoral bowing (Table 2). This trend was consistent regardless of osteotomy method or derotation angle, indicating a strong relationship between sagittal bowing and coronal alignment change.

**Table 2:** mLDFA change after supracondylar FDO based on femoral antecurvature, osteotomy method and derotation angle.

Osteotomy method		Group						
	Derotation angle	1		2		3		*p*‐Value
Shaft‐perpendicular osteotomy	10°	0.5 (1.5–0.6)		0.9 (0.8–1.0)		1.5 (1.3–1.6)		<0.001^a^
	20°	1.3 (1.1–1.4)		2.0 (1.9–2.3)		3.1 (2.8–3.4)		<0.001^a^
	*p*‐Value^b^	<0.001		<0.001		<0.001		
DFC‐parallel osteotomy	10°	0.4 (0.3–0.5)		0.8 (0.7–0.8)		1.2 (1.2–1.3)		<0.001^a^
	20°	0.8 (0.6–0.9)		1.6 (1.4–1.6)		2.4 (2.3–2.6)		<0.001^a^
	*p*‐Value^b^	<0.001		<0.001		<0.001		
*p*‐Value		<0.001^c^	<0.001^d^	<0.001^c^	<0.001^d^	<0.001^c^	<0.001^d^	

*Note*: Values are expressed as median with interquartile range (Q1–Q3) in parentheses.

Abbreviations: FDO, femoral derotational osteotomy; mLDFA, mechanical lateral distal femoral angle.

^a^ Comparison by group within each cutting plane and rotation using Kruskal–Wallis test. Bonferroni method showed statistically significant difference between all three groups.

^b^ Comparison by rotation within each group and cutting plane using Wilcoxon signed‐rank test.

^c^ Comparison by cutting plane within each group and 10° rotation using Wilcoxon signed‐rank test.

^d^ Comparison by cutting plane within each group and 20° rotation using Wilcoxon signed‐rank test.

### Combined effects of derotation angle and osteotomy method

The amount of derotation and orientation of the osteotomy significantly influenced mLDFA change (Table 2). Increasing the derotation angle from 10° to 20° significantly increased the valgus shift in all three groups (Wilcoxon signed‐rank test, *p* < 0.001). On the contrary, DFC‐parallel osteotomy consistently reduced the magnitude of valgus shift compared to shaft‐perpendicular osteotomy. Multivariable linear regression analysis confirmed these effects (Table 3). The interaction between femoral antecurvature and derotation angle was statistically significant (*β* = 0.015, *p* < 0.001), indicating amplification of the valgus‐inducing effect of derotation with greater femoral bowing. In contrast, the interaction between femoral antecurvature and osteotomy method was not significant (*β* = −0.005, *p* = 0.759), indicating a consistent protective effect of performing DFC‐parallel osteotomy (*β* = −0.331, *p* = 0.005) across all degrees of antecurvature.

**Table 3:** Multivariable linear regression analysis of mLDFA change: overall model with interaction effects between DDA, derotation angle and osteotomy method.

Variable	Coefficient (*β*)	Standard error	*t*	*p* value	95% confidence interval	
					Low	High
Intercept	0.065	0.192	0.337	0.736	−0.313	0.442
DDA	0.003	0.026	0.101	0.92	−0.048	0.054
Derotation angle	−0.005	0.012	−0.436	0.663	−0.028	0.018
Osteotomy method	−0.331	0.116	−2.860	0.005	−0.558	−0.103
DDA | Derotation angle	0.015	0.002	9.405	<0.001	0.012	0.018
DDA | Osteotomy method	−0.005	0.016	−0.307	0.759	−0.036	0.026

Abbreviations: DDA, distal diaphyseal angle; mLDFA, mechanical lateral distal femoral angle.

### Proposed strategy for minimising valgus shift

**Figure 3:**
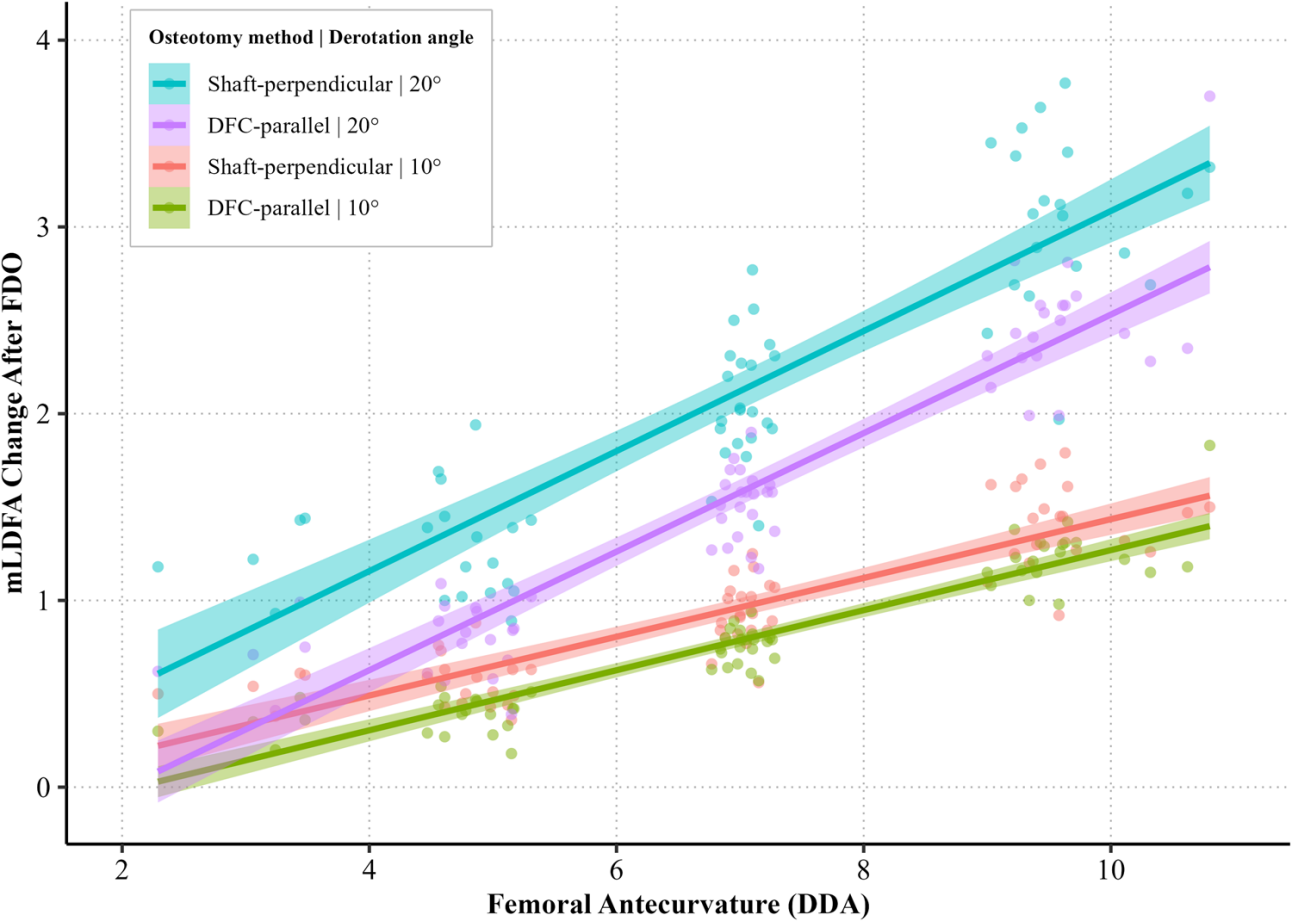
Multivariable linear regression analysis of mechanical lateral distal femoral angle change for each combination of osteotomy method and derotation angle.

**Figure 4:**
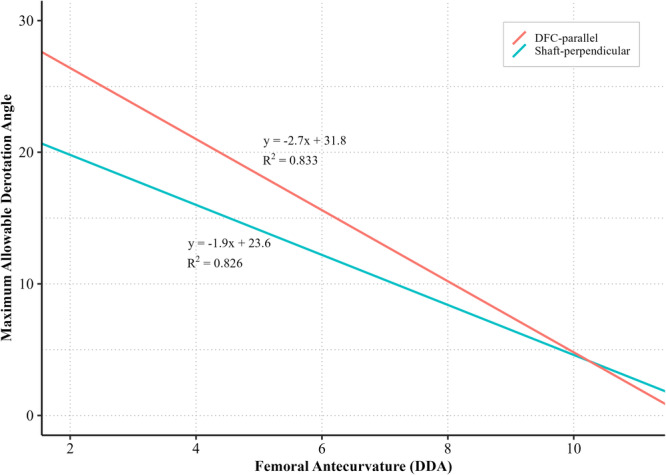
Predicted maximum allowable derotation angle based on femoral antecurvature. A shaft‐perpendicular cutting method (blue line) and a distal femur condylar (DFC)‐parallel cutting method (red line) are compared. For lower distal diaphyseal angle (DDA) values, DFC‐parallel osteotomy allows greater derotation while maintaining valgus shift <1°. As DDA increases beyond approximately 10°, the allowable derotation decreases rapidly for both techniques.

The most effective strategy for minimising valgus shift after supracondylar FDO was a combination of DFC‐parallel osteotomy with 10° of derotation, particularly in femurs with greater sagittal bowing. Based on stratified regression analysis performed for each cutting method and derotation angle (Figure 3 and Table 4), DFC‐parallel osteotomy with 10° derotation demonstrated the lowest slope (*β* = 0.147) and highest *R*² value (0.84), indicating minimal sensitivity to sagittal bowing and excellent predictability. In addition, greater derotation was tolerable in femurs with lesser anterior bowing, whereas femurs with higher antecurvature required more conservative correction to avoid mLDFA change >1° (Figure 4).

**Table 4:** Multivariable linear regression analysis of mLDFA change for each combination of osteotomy method and derotation angle.

Osteotomy method | Derotation angle	Intercept	Slope (*β*)	95% confidence interval	*p*‐Value	*R* ^2^
Shaft‐perpendicular | 10°	−0.063	0.147	0.126–0.168	<0.001	0.755
Shaft‐perpendicular | 20°	0.041	0.298	0.256–0.341	<0.001	0.755
DFC‐parallel | 10°	−0.239	0.147	0.131–0.163	<0.001	0.840
DFC‐parallel | 20°	−0.444	0.289	0.257–0.321	<0.001	0.835

Abbreviations: DFC, distal femur condylar; mLDFA, mechanical lateral distal femoral angle.

## DISCUSSION

This study investigated the influence of femoral sagittal bowing, derotation angle and osteotomy orientation on coronal alignment change after supracondylar FDO using 3D CT‐based simulation. The principal findings were threefold: (1) greater femoral antecurvature resulted in a more pronounced valgus shift after supracondylar FDO; (2) larger derotation angles exacerbated this effect, particularly in femurs with greater bowing, whereas DFC‐parallel osteotomy consistently mitigated coronal malalignment and (3) making DFC‐parallel osteotomy combined with 10° of derotation offered the most favourable profile for minimising valgus change, especially in femurs with high anterior bowing.

In group‐based comparison, higher femoral antecurvature resulted in more pronounced valgus shift after supracondylar FDO regardless of derotation amount and osteotomy method. Nelitz et al. reported similar mLDFA reduction with supracondylar FDO, with greater changes occurring in more highly derotated and antecurved simulated femurs [11]. Konrads et al. also demonstrated valgus changes in clinical supracondylar FDO cases, attributing this change to reorientation of the femoral antecurvature [9]. The present study reinforces this association using cadaveric femoral models. This unintended change in coronal alignment can be attributed to geometric interplay between the femoral antecurvature and the axis of rotation. When the femur bows anteriorly, axial rotation creates a secondary component of motion in the coronal plane. Lee et al. described this phenomenon in their study and raised awareness of unexpected angular deformity after surgery involving osteotomy [10]. The observed valgus shift tendency, which was exacerbated in the increased femoral bowing group, emphasises the importance of preoperative evaluation of such a shift for recurrent patellar dislocation in patients undergoing supracondylar FDO.

The amount of derotation was associated with valgus change after supracondylar FDO. In group‐based analysis, de‐rotation of 20° showed greater reduction in mLDFA than 10° in all three groups, regardless of the osteotomy method. According to multivariable linear regression, there was an interaction between femoral bowing and derotation amount, with greater anterior bowing associated with a more pronounced valgus shift after a high degree of derotation. Nelitz et al. also simulated derotation angles of 10°, 20° and 30° and reported more pronounced valgus shift with increasing external derotation [11]. However, their study did not evaluate the interaction between derotation angle and femoral antecurvature. The present study expands on this by integrating both variables in a regression model. Currently, there is no clear guideline on the target degree of femoral anteversion or the optimal amount of derotation during supracondylar FDO [17]. The present study's findings show that the effect of derotation on valgus tendency depends on the degree of femoral antecurvature, which may offer further insights into the appropriate amount of derotation during supracondylar FDO. Osteotomy orientation also played a critical role, as the DFC‐parallel osteotomy consistently reduced valgus shift across all bowing levels. Although Flury et al. showed minimal alignment change when osteotomies were performed perpendicular to the mechanical axis in both planes, such precision is practically not possible intraoperatively [2]. During surgery, only a limited view of the distal femur can be visualised with intraoperative fluoroscopy. Therefore, adjusting the osteotomy plane to be perpendicular to the mechanical axis is challenging, especially in the sagittal plane. However, coronal control can be achieved by using the distal femur condylar line as a reference. The results of the present study suggest that this simple intraoperative adjustment can have a substantial effect on coronal alignment.

When combining all variables, the safest and most effective method was DFC‐parallel osteotomy with 10° of derotation. Stratified regression confirmed that this combination had the lowest DDA sensitivity and the best model fit. Furthermore, the maximum allowable derotation angle to stay within a 1° valgus shift was higher when performing DFC‐parallel osteotomy (Figure 4). This allows the surgeon to individualise the osteotomy strategy based on preoperative assessment of femoral antecurvature. Several efforts have been made to minimise coronal malalignment after FDO. Nelitz et al. performed osteotomy at the mid‐shaft rather than the supracondylar area to achieve the smallest change in coronal alignment [11]. However, the choice of osteotomy at the mid‐shaft level in adults is difficult due to the increased risk of nonunion. Imhoff et al. conducted several studies to reduce valgus shift after supracondylar FDO and suggested a mathematical model, the Pillar‐Crane model, to minimise unintended valgus change [5]. The authors validated this method in a cadaveric femur with a defined cutting angle calculated using the proposed mathematical model [4]. In their study, the desired coronal alignment after supracondylar FDO was achieved using their model‐based cutting angle and a 3D‐printed cutting guide [6]. Neopoulos et al. used a patient‐specific instrument (PSI) to correct both rotational and coronal alignments [12]. These methods, although biomechanically sound, require extensive planning and resources. In contrast, the strategy proposed in this study is simple and easily applicable within standard intraoperative workflows using a guidewire and intraoperative fluoroscopy.

The present study has several limitations. First, the analysis was based on cadaveric CT data, which did not account for soft tissue constraints or dynamic muscle forces that may influence actual surgical outcomes. Second, among morphological variables, only sagittal femoral bowing was considered. Other factors, such as the neck‐shaft angle, were not evaluated and should be investigated in future studies. Third, the mean mLDFA in our sample was lower than the values typically reported in other studies. While this discrepancy may reflect the ethnic variation in distal femoral morphology, the lower baseline mLDFA may have amplified the magnitude of valgus shift after FDO. Therefore, caution should be warranted to generalise our findings to the general population. Fourth, although cross‐checked, the data were collected by a single investigator. Finally, the threshold of 1° for mLDFA change was arbitrarily selected, and its long‐term clinical significance remains uncertain.

## CONCLUSION

Femoral antecurvature significantly affects coronal alignment after supracondylar FDO. Although the optimal target angle for derotation remains a surgical choice, understanding the relationship between sagittal bowing and coronal alignment can help tailor patient‐specific decisions. To minimise valgus shift after supracondylar FDO, aligning the osteotomy plane parallel to the distal femur condylar line can be effective. This method is simple and practical for standard surgical procedures.

## AUTHOR CONTRIBUTIONS

Jisu Park and Hyunkwon Kim drafted the manuscript. Jisu Park, Hyunkwon Kim and Seonjin Shin constructed the database and conducted data analysis. Tae Woo Kim, Moon Jong Chang and Dai‐Soon Kwak contributed to the design of the study. Jisu Park, Hyunkwon Kim and Dai‐Soon Kwak contributed to the interpretation of the data. Dai‐Soon Kwak revised the manuscript. All authors read and approved the final manuscript.

## CONFLICT OF INTEREST STATEMENT

The authors declare no conflicts of interest.

## ETHICS STATEMENT

The authors have nothing to report.

## Data Availability

The datasets used and/or analysed during the current study are available from the corresponding author on reasonable request.
